# Use of magnetic source imaging to assess recovery after severe traumatic brain injury—an MEG pilot study

**DOI:** 10.3389/fneur.2023.1257886

**Published:** 2023-11-03

**Authors:** Anand Karthik Sarma, Gautam Popli, Anthony Anzalone, Nicholas Contillo, Cassandra Cornell, Andrew M. Nunn, Jared A. Rowland, Dwayne W. Godwin, Laura A. Flashman, Daniel Couture, Jennifer R. Stapleton-Kotloski

**Affiliations:** ^1^Department of Neurology, Wake Forest University School of Medicine, Winston-Salem, NC, United States; ^2^Neurocritical Care, Piedmont Atlanta Hospital, Atlanta, GA, United States; ^3^Wake Forest University School of Medicine, Winston-Salem, NC, United States; ^4^Department of Neurosurgery, Henry Ford Health System, Detroit, MI, United States; ^5^Department of Surgery, Wake Forest University School of Medicine, Winston-Salem, NC, United States; ^6^Department of Neurobiology and Anatomy, Wake Forest University School of Medicine, Winston-Salem, NC, United States; ^7^Research and Education Department, W.G. (Bill) Hefner VA Healthcare System, Salisbury, NC, United States; ^8^Department of Neurosurgery, Wake Forest University School of Medicine, Winston-Salem, NC, United States

**Keywords:** severe TBI, MEG, coma, longitudinal, recovery, synthetic aperture magnetometry, source localization

## Abstract

**Rationale:**

Severe TBI (sTBI) is a devastating neurological injury that comprises a significant global trauma burden. Early comprehensive neurocritical care and rehabilitation improve outcomes for such patients, although better diagnostic and prognostic tools are necessary to guide personalized treatment plans.

**Methods:**

In this study, we explored the feasibility of conducting resting state magnetoencephalography (MEG) in a case series of sTBI patients acutely after injury (~7 days), and then about 1.5 and 8 months after injury. Synthetic aperture magnetometry (SAM) was utilized to localize source power in the canonical frequency bands of delta, theta, alpha, beta, and gamma, as well as DC–80 Hz.

**Results:**

At the first scan, SAM source maps revealed zones of hypofunction, islands of preserved activity, and hemispheric asymmetry across bandwidths, with markedly reduced power on the side of injury for each patient. GCS scores improved at scan 2 and by scan 3 the patients were ambulatory. The SAM maps for scans 2 and 3 varied, with most patients showing increasing power over time, especially in gamma, but a continued reduction in power in damaged areas and hemispheric asymmetry and/or relative diminishment in power at the site of injury. At the group level for scan 1, there was a large excess of neural generators operating within the delta band relative to control participants, while the number of neural generators for beta and gamma were significantly reduced. At scan 2 there was increased beta power relative to controls. At scan 3 there was increased group-wise delta power in comparison to controls.

**Conclusion:**

In summary, this pilot study shows that MEG can be safely used to monitor and track the recovery of brain function in patients with severe TBI as well as to identify patient-specific regions of decreased or altered brain function. Such MEG maps of brain function may be used in the future to tailor patient-specific rehabilitation plans to target regions of altered spectral power with neurostimulation and other treatments.

## 1. Introduction

Globally, 69 million individuals sustain a traumatic brain injury each year, and of those, 5.48 million are estimated to have sustained a severe traumatic brain injury (sTBI) (1), although TBI events are likely underreported worldwide (2). The Centers for Disease Control estimated that 2.87 million individuals sustained an sTBI in 2014 (3), the most recent report for the United States. According to the TBIMS National Database (4), 5-year outcomes for patients ages 16 and older with sTBI who receive inpatient rehabilitation include a mortality rate of 23%, a 30% chance of worsening, a 22% chance of remaining the same, and a 26% chance of improvement. Thus, methods to identify accurate patient prognoses are critically needed.

Early comprehensive neurosurgical and neurocritical care are recommended in patients with sTBI (5) and result in better outcomes (6, 7). However, even with the resources of an American College of Surgeons verified Level I Trauma Center, sTBI can prove devastating and often the medical team and the patient’s family must decide whether to continue aggressive care in the anticipation that the patient will survive with a reasonable quality of life or to withdraw life support (8). An ideal prognostic model would permit those involved to unite estimates of functional recovery with decision-making about personalized interventions.

Following acute sTBI, patients are comatose, a period characterized by unresponsiveness and a lack of wakefulness and awareness (9). Prolonged disorders of consciousness (DoC) generally last more than 28 days and encompass patients in the vegetative state/unresponsive wakefulness state (VS/UWS), or in the minimally conscious state (MCS) (10). Patients within the VS/UWS state demonstrate wakefulness without awareness, while patients within the MCS state exhibit impaired responsiveness, evidence of limited awareness, and the ability to follow simple commands or generate purposeful behavior (9). Misdiagnosis rates for patients with DoC are unacceptably high; ~40% of patients misclassified as in VS/UWS state were actually in MCS (10–12). Furthermore, patients with sTBI may regain consciousness prior to regaining the ability for self-expression (13–15), but without behavioral evidence of awareness tragically these patients may receive an incorrect prognosis and be denied either critical life support (8) or rehabilitation (16).

Prior efforts to predict survival and disability outcomes have resulted in the development of prediction models such as the International Mission for Prognosis and Analysis of Clinical Trials in TBI (IMPACT) and Corticosteroid Randomization After Significant Head Injury (CRASH) (17, 18). While both have externally been shown to demonstrate validity and reliability (19–22) it has been proposed that scoring systems such as CRASH and IMPACT are not exacting enough to assist in informing decisions at the individual patient level (23, 24). Most prognostic tools fail to incorporate complex and unique considerations of each patient such as pre-injury morbidity, mechanism of injury, or complex pathophysiological findings [e.g., hemorrhage vs. edema vs. contusion vs. diffuse axonal injury (DAI)]. Thus, physicians care for these patients with limited prognostic resources but, nonetheless, must aid families in making decisions about continuation of care and expectations of recovery. To better assist prognostic efforts, a more applicable and personalized predictive instrument could prove useful.

A patient-oriented quantitative assessment of neurological activity may assist in providing a diagnosis and predicting recovery from sTBI. Indeed, fMRI has revealed evidence of language processing (15, 25–27) and covert command following (14, 16, 28) in some patients with DoC, thus providing evidence of a higher level of awareness and cognitive function than revealed by bedside behavioral testing. Similarly, PET has been used to distinguish levels of consciousness on the basis of whole brain metabolism (29), to localize the extent of brain network dysfunction (30), to identify islands of function (31), and to correlate metabolism, electrical activity, and cognitive state (32). Likewise, EEG resting state rhythms (28, 33–35), spectral characteristics (32, 36–39), quantitative EEG (40–42), and microstates (43) can be linked to the extent of brain damage and to levels of consciousness, although EEG findings can be heterogenous despite DoC level (44). Finally, multimodal techniques have indicated that resting state network connectivity (37, 45, 46), and specifically, alterations or improvements in default mode network connectivity (47), correlate with levels of consciousness (48–50) and with recovery trajectories (51, 52).

Magnetoencephalography (MEG) is a neurophysiological technique for measuring the biomagnetic fields directly generated by brain activity (53). MEG has been available as a clinical and research tool for decades (54–56), used primarily for the localization of epileptic foci and for presurgical functional brain mapping (57–60). MEG confers several advantages over other modalities such as fMRI, PET, and EEG since it is a direct measure of brain activity, it possesses excellent spatial and temporal resolution (61), and, because the body is magnetically transparent, MEG signals are not distorted by the resistive qualities of the head. Finally, through the use of magnetic source imaging (MSI), biomagnetic activity can be directly localized in brain space (58, 62). Despite these advantages, the use of MEG in TBI research has focused predominantly on mild or moderate TBI (56, 63–70), with only two reports on patients with severe brain injury (31, 71).

In this study, we aim both to examine the feasibility of conducting MEG scans shortly after injury as well as to evaluate brain function over time using source-localized, resting state MEG maps as an adjunct to individualized standard-of-care protocols, and as a comparison to patient presentation and clinical assessment of cognitive function. Outcomes will inform the potential value of including these measures as part of a comprehensive evaluation following sTBI and will also inform a larger prospective study of MEG for sTBI prognostication.

## 2. Materials and methods

### 2.1. Patients

This project was reviewed and approved by the institutional review board at Atrium Health Wake Forest Baptist Medical Center in Winston-Salem, North Carolina. The welfare and privacy of human subjects were protected. Informed consent was provided by legally authorized representatives as the patients were comatose at the time of entry into the study. Adult patients (>18 years, *n* = 7) who had experienced blunt force trauma [falls, motor vehicle collision (MVC), etc.] resulting in sTBI [Glasgow Coma Scale (GCS) score 3–8] and admitted to the Trauma Unit were screened and enrolled in the study > 7 days since injury. Exclusion criteria encompassed patients with prior severe brain injury, neurodegenerative disease, penetrating TBI with or without intracranial metallic foreign bodies precluding MRI, pregnant females, prisoners, patients in custody or psychiatric hold, major debilitating baseline mental health disorders that would interfere with follow-up and validity of outcome assessments, spinal cord injury of American Spinal Injury Association score C or worse, concomitant severe polytrauma, and individuals being screened for death by neurological criteria.

Patients underwent MRI scans first and then MEG scans (~7 days after injury) when deemed sufficiently stable. A neurocritical care physician, registered nurse, and respiratory therapist were present during transport and for the duration of the scans. Ventilators, intravenous infusion pumps, external ventricular drains, and other electrical or battery-operated devices were stationed outside the rooms housing the MRI and MEG for safety and to prevent artifact. Life support equipment including oxygen sources and emergent cardiopulmonary resuscitation supplies were also available.

**Figure 1:**
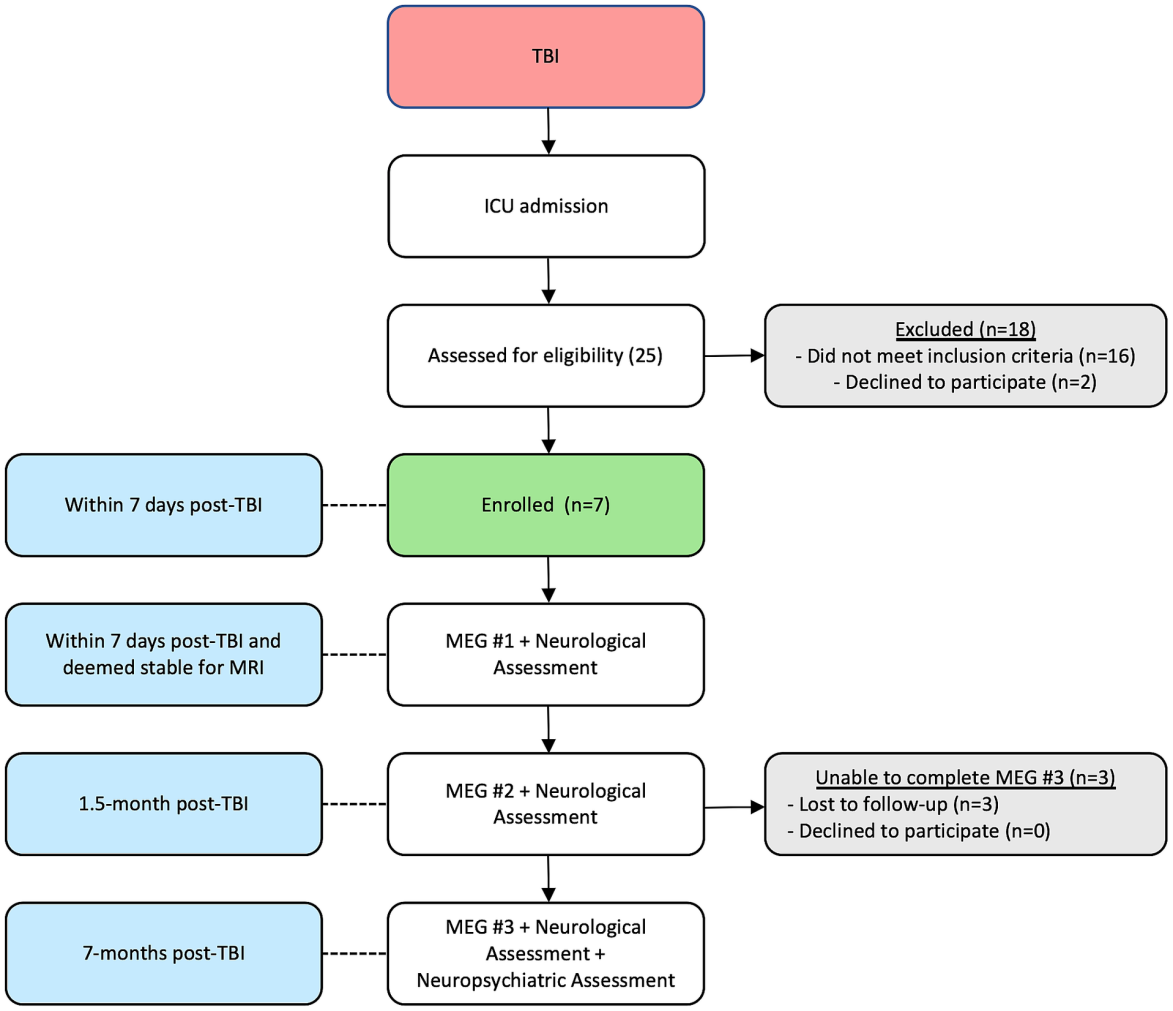
Timeline of events and enrollment outcomes.

Patients returned for MEG scans, neurological assessments, and clinical outcome measures/scores at ~1.5- and 8-months post injury. No patient required life support for the second and third scans. At the third scan returning patients were ambulatory and also received a 90-min neuropsychological screening battery, assessing domains such as baseline functioning, verbal memory, auditory attention and working memory, information processing speed, response inhibition, and mood assessment including depression and anxiety (see Figure 1 for the timeline of events and enrollment patterns). Glasgow Outcome Score-Extended (GOSE) were also obtained for the four returning patients.

MEG data from 30 healthy Veteran participants were obtained from a separate study [Chronic Effects of Neurotrauma Consortium Study 34 (56, 63–65)] and were used as a control group for the current study. In this study, 8 mins of resting state data were acquired in the seated position with eyes open. The Veteran participants were previously deployed, had no history of TBI, posttraumatic stress disorder, severe mental health disorder, nor current substance use disorder. Their demographics were as follows: 23 males; 25 right hand dominant; average age 43.0 years (SD = 10.3, range 30–71 years), average IQ 101.2 (SD = 12.7, range 81–119), and average years educated 15.6 (SD = 2.7, range 12–22 years).

### 2.2. MEG recordings

Data were acquired using a whole-head CTF Systems Inc. MEG 2005 neuromagnetometer system equipped with 275 first-order axial gradiometer coils and 29 reference sensors. Head localization was achieved using a conventional three-point fiducial system (nasion and preauricular points). Resting-state recordings (5–10 min) were conducted in the supine position with eyes open when the patients were awake, and eyes closed when they were not. Simultaneous EEG was recorded with whole-scalp coverage using the International 10–20 system of electrode placement. Both MEG and EEG were sampled at 1,200 Hz over a DC-300 Hz bandwidth.

### 2.3. MEG analysis

All preprocessing and beamforming were performed in the CTF MEG™ Software package (CTF MEG Neuro Innovations, Inc., Coquitlam, BC, Canada). MEG data were pre-processed offline using synthetic third-order gradient balancing, whole trial DC offsetting, and band pass filtering from DC-80 Hz with a 60 Hz notch filter. Data were visually inspected for muscle artifact, and such epochs were discarded from further analyses. No other artifact rejection was conducted because beamforming automatically performs excellent noise suppression (72–76). A well-validated beamformer (synthetic aperture magnetometry; SAM) (77, 78) was applied (voxel size of 5 × 5 × 5 mm) using a multiple local spheres head model based on the participant’s MRI (79) to construct single-state, noise-normalized, volumetric, statistical parametric maps, identifying areas of significant brain activity for each participant individually. The SAM Ƶ-score is a ratio of source power to noise variance for a given voxel that is analogous to the z-deviate (77, 80). SAM was applied in the following frequency ranges: delta (DC–4 Hz), theta (4–8 Hz), alpha (8–13 Hz), beta (13–30 Hz), and gamma (30–80 Hz), as well as DC–80 Hz (56, 63, 64). The magnitudes and coordinates for all local Ƶ-score maxima, or peaks, in each frequency map were extracted. The local maxima are voxels whose values are greater than all neighboring voxels in the map and possess a high signal to noise ratio. SAM peaks have been shown to colocalize to significant sources, or generators, of neurophysiological activity (77, 80, 81), and each frequency map may contain multiple local maxima due to the pattern of underlying brain activity (e.g., Supplementary Figure 1).

### 2.4. Anatomic localization of SAM sources

All SAM maps and corresponding MRIs were imported into Matlab 2020a (Natick, Massachusetts: The MathWorks Inc., RRID:SCR_001622) and analyzed with the FieldTrip toolbox (https://www.fieldtriptoolbox.org, RRID:SCR_004849) (82). The MRI and SAM maps for each patient were transformed into MNI space and co-registered to the AAL atlas (83). Additionally, the cortical surface mesh was extracted from each MRI using FreeSurfer (http://surfer.nmr.mgh.harvard.edu, RRID:SCR_001847) (84) and down sampled with Connectome Workbench (https://www.humanconnectome.org/software/connectome-workbench, RRID:SCR_008750) (85). Custom scripts based on the FieldTrip toolbox were used to plot the coregistered MRI and SAM maps as axial slices, as volumetric glass brains, or as surface-level maps, the last on the extracted cortical meshes. Given the diversity of injury types, and to better illustrate the patterns of activation and deactivation, the displays of the SAM maps were scaled individually for each patient and each frequency band, but the plotting range across scan timepoints was scaled according to the maximal Ƶ-score within each frequency band. The scaling range for each patient was maintained across the different plot types (slice, surface, or glass).

### 2.5. Statistics

SAM can resolve sources separated by 500 μm (78, 86) as well as deep sources, even under difficult imaging conditions (61, 72). SAM peaks correspond to significantly active neural sources (80, 81), and voxels that fall within the full width half max around the peak voxel are not significantly different. Thus, to identify which neural generators were active at a given scan time point (acute, 1.5-month post-injury, and 8-month post-injury) and in which frequency bands, the Ƶ-score of the peak voxels in each patient’s maps were extracted.

Many regions of interest (ROIs, as defined by the AAL parcellation) lacked local maxima at a given frequency band. Part of the reason for this is that different brain regions have different resting state frequencies and may exhibit a peak in one frequency band and not in others. A second reason for a lack of peaks in a given ROI could be that regional activity might be contiguous with the FWHM of a peak in another ROI, and the largest voxel value in the former ROI would not actually be a local maximum in the whole brain map itself. To assess the global activity level in an ROI without a peak, the median SAM voxel Ƶ-score was used as an assumption-free metric. This resulted in a vector of Ƶ-score (either peak or median values), with one value per ROI for each frequency band, and on a per subject basis.

To examine the potential hemispheric asymmetry in neurological activity for each patient or control participant, the peak or median Ƶ-score values of the left hemispheric ROIs were subtracted from their matching right hemispheric ROI values for each frequency band. This difference was then divided by the average of the activity in each ROI pair and expressed as a percent. These percent differences were calculated for each scan time point for each patient and plotted in a bar graph to track changes in each ROI over time as well as to compare against the values for control participants (Supplementary Figures 2, 3).

Group-wise changes according to frequency band and scan time were assessed. For each patient, the total number of peaks across all ROIs was calculated for each frequency band and scan time point, and group patient means per bandwidth were calculated for each scan timepoint. Group means at each bandwidth were also calculated for the control participants. This enabled a quantification of the number of neural generators operating at a particular frequency and whether this changed over scan time points, and how the number of neural generators at each scan compared to the control group. Secondly, for each patient or control participant, the peak Ƶ-score power was averaged across all ROIs and was calculated for each frequency band and scan time. Group patient means per bandwidth were calculated for each scan timepoint, and group means at each bandwidth were also calculated for the control participants. These means enabled a quantification of the power at a particular frequency and whether this changed over scan time points, and how such power compared to the control group at each scan. Scans in which the patient’s head was not fully inserted into the helmet were discarded from the group analyses due to incomplete estimates of the number of neural generators and their potential power. Two multiple linear regression models were constructed in R version 4.2.0, “Vigorous Calisthenics,” RRID:SCR_001905 (87), with the first modeling the number of peaks for each patient or control participant as a function of scan condition (scan 1, scan 2, scan 3, or control), bandwidth (delta, theta, alpha, beta, gamma, full bandwidth), and the interaction of scan condition and bandwidth. The second modeled the Ƶ-score power for each patient or control participant as a function of scan condition (scan 1, scan 2, scan 3, or control), bandwidth, and the interaction between scan condition and bandwidth. All factors were modeled as indicators, and the number of peaks and the Ƶ-score power were modeled as continuous variables. Given significant interaction terms in both models, simple effects contrasts were conducted within each bandwidth to determine if the peak power and the number of peaks differed between the scan timepoints as contrasted to the values for the control participants. Simple effects contrasts were conducted with the phia package version 0.2–1 in R (88). The Holm method was used to control the family-wise error rate (FWER) at an overall alpha of 0.05; this method is equivalent to the Bonferroni method in controlling Type I error but is better at controlling the Type II error rate (89).

## 3. Results

Seven patients with sTBI and admitted to the Trauma ICU were enrolled in the study. Table 1 describes the patient demographics, including etiology and mechanism of injury. All patients were intubated and on artificial ventilation at the time of initial assessment, enrollment, the first MEG scan, and the MRI. None were on any sedative or analgesic infusions, including propofol, benzodiazepine, or opioids for at least a 24-h period prior to the scan. Weaning and maintaining patients off sedatives and analgesics while still intubated and on mechanical ventilation is common practice in patients recovering from coma and encephalopathy. While beneficial to MEG and EEG recordings, this was not a deviation from standard-of-care clinical practice for the purpose of the study. First MEG scans were performed at a mean ± SD time of 6.6 ± 4.3 days after injury. Mean time to scan 2 after injury was 47.6 ± 20.6 days, and for scan 3 was 7.8 ± 1.5 months. All patients completed scans 1 and 2, while only four returned for scan 3. At the third scan, all patients were awake and ambulatory.

**Table 1:** Patient demographics, injury characteristics, and Glasgow Coma Scale (GCS) progression at each scan time point.

Patient ID	Age (y)	Sex	Dominant hand	Injury mechanism	TBI Type	LOC	AIS (head/neck)	Adm GCS	Scan 1 GCS	Scan 2 GCS	Scan 3 GCS	GOSE
1	43	M	Right	MVC	Closed	Yes	4	8	12	15	15	7
2	53	M	Right	MVC	Closed	Yes	5	10	10	15	-	-
3	56	M	Right	Fall from height	Closed	Yes	5	5	9	13	-	-
4	54	M	Right	Blunt trauma	Crush	Yes	5	3	12	15	15	7
5	71	F	Right	MVC	Closed	No	5	9	9	13	-	-
6	26	M	Right	MVC	Closed	Yes	5	9	9	15	15	6
7	28	M	Left	MVC	Closed	Yes	5	7	7	12	15	7

y, years; M, male; F, female; MVC, motor vehicle crash; LOC, loss of consciousness; AIS, abbreviated injury scale; Adm, admission; GOSE, Glasgow Outcome Scale-Extended.

As a group, the patients had problems with mood (depression/anxiety) and also had deficits in attention and response inhibition at scan 3. The small sample size precluded direct correlation with MEG, but the functional findings confirmed deficits we hope to link to the MEG findings in a future study.

### 3.1. Patient 1

**Figure 2:**
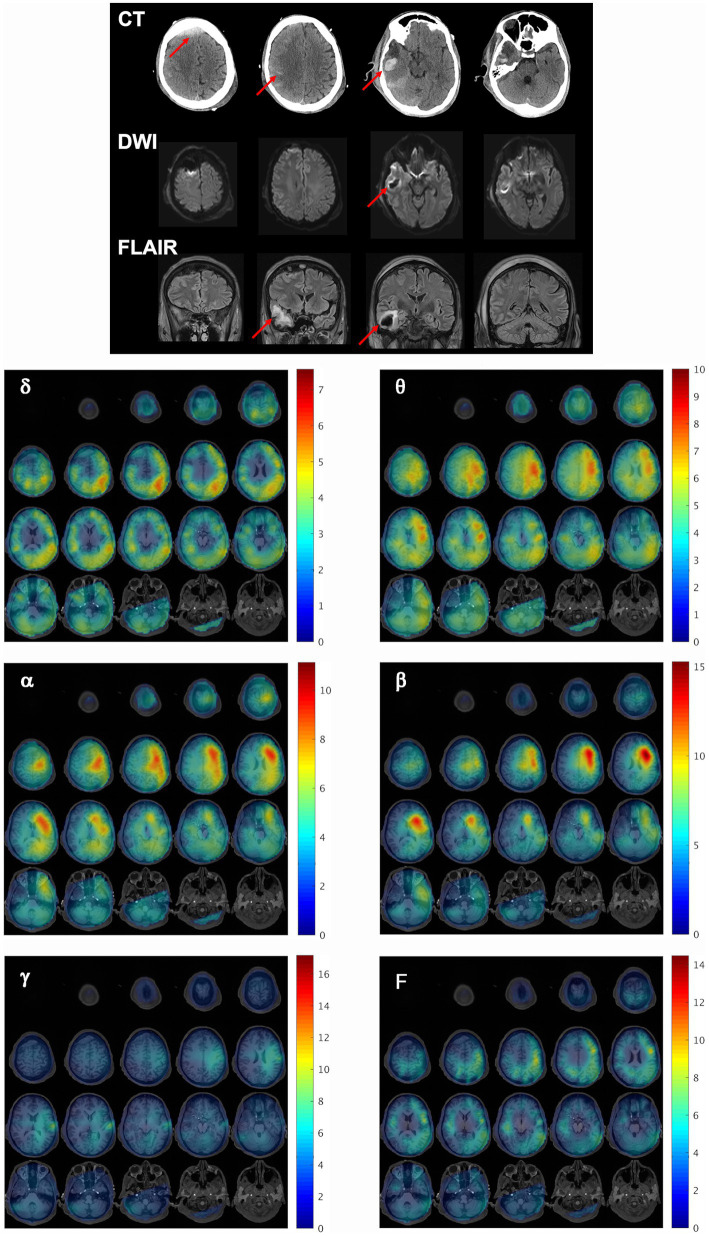
Structural and functional findings shortly after injury. (Top) Representative CT, DWI, and FLAIR slices reveal extensive right frontal and temporal lesions in patient 1, 2 days after initial injury. Red arrows indicate regions of injury. (Bottom) MEG synthetic aperture magnetometry volumetric maps were constructed for delta (δ), theta (θ), alpha (α), beta (β), gamma (γ), and full (F, DC-80 Hz) bandwidths and overlaid on the patient’s MRI to localize resting state brain activity at the first scan timepoint. The activity at each frequency band is depicted as a series of axial slices with the corresponding color bar to the right of each grid indicating the Ƶ-score range for the voxels in the SAM map. All slices are presented in radiological coordinates. The Ƶ-score color bar range is maintained the same across all scan time points within each frequency band for subsequent figures for this patient to facilitate the visualization of changes across time.

Patient 1 was used as the model patient to highlight the MEG results. Patient 1 was a 43-year-old (y.o.), right-handed male with a past medical history (PMH) of type-2 diabetes mellitus, Crohn’s disease, and depression, who was admitted after being struck as a pedestrian by a motor vehicle. His injuries included a calvarial fracture, intraparenchymal hemorrhage (IPH), hemorrhagic contusion of the anterior right temporal lobe, high right frontal epidural hematoma (EDH) with extension across the midline, right frontal subdural hematoma (SDH), scattered trace right convexity subarachnoid hemorrhage (SAH), and fractures of the right first and fifth phalanx, left 6^th^ rib, and left medial malleolus. Extensive right frontal and temporal lesions were evident on CT, DWI, and FLAIR (Figure 2, top).

This patient received his first MEG scan 2 days after injury. Figure 2 (bottom) demonstrates whole brain maps of biomagnetic activity for delta (δ), theta (θ), alpha (α), beta (β), gamma (γ), and full bandwidth (F, DC-80 Hz) oscillations overlaid on the patient’s MRI. The activity at each frequency band is depicted in a series of axial slices with the corresponding color bar to the right of each grid indicating the Ƶ-score range for the voxels in the SAM map. For all bandwidths, brain activity was severely reduced in the entire right hemisphere relative to the left, and in both hemispheres, gamma power was minimal. Supplementary Figure 2 depicts the percent difference in activity for pairs of ROIs for each bandwidth across the scan timepoints. The fourth column depicts the average percent difference for the control participants, who tended to have slightly more power (approximately less than 25%) in left than right ROI pairs across bandwidths, likely because most were right-handed. In contrast, for patient 1 at the first scan many ROI pairs had 50% more activity on the left than the right, and some exceeded 100%.

Patient 1 was later discharged to the rehabilitation floor and returned for his second MEG scan 57 days after injury, off artificial ventilation, and with eyes open during the scan. He was eventually discharged and returned for a follow-up visit at 6 months post injury for a third scan along with neurological assessment and neuropsychological testing, the latter of which showed evidence of depression and problems with response inhibition.

**Figure 3:**
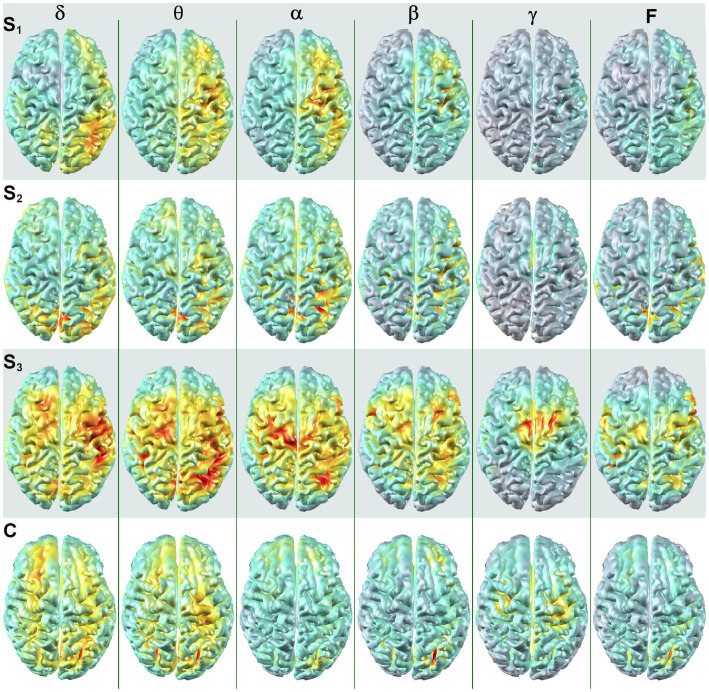
SAM surface maps of brain activity change across scan time points. Each column depicts the SAM SPMs overlaid on the dorsal cortical surface of the brain for patient 1 for each frequency band. Each row depicts a set of maps at a given time point, with S_1_ indicating activity during the first MEG scan, S_2_ indicating activity for the second MEG scan, and S_3_ indicating activity for the third MEG scan. A single control participant (C) is presented in the bottom row, and the control maps are on the same color scale as the maps for the patient. Conventions are the same as in Figure 2. All brain volumes are in radiological coordinates.

**Figure 4:**
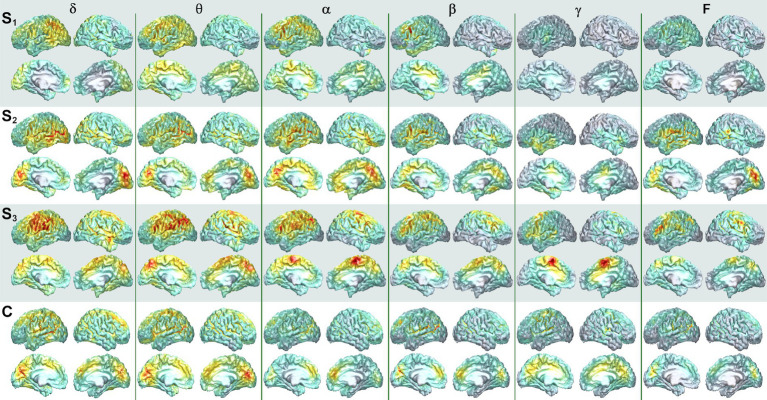
Mesial and lateral SAM surface maps of brain activity changes. Each column depicts the SAM SPMs overlaid on the left and right, lateral and mesial, cortical surfaces of the brain for patient 1 for each frequency band (columns). The left hemisphere is presented on the left for this figure. Each pair of rows depicts a set of maps at a given scan time point, with scan number denoted by S_1_, S_2_, and S_3_. Similar surface maps are presented for the representative control participant (C).

**Figure 5:**
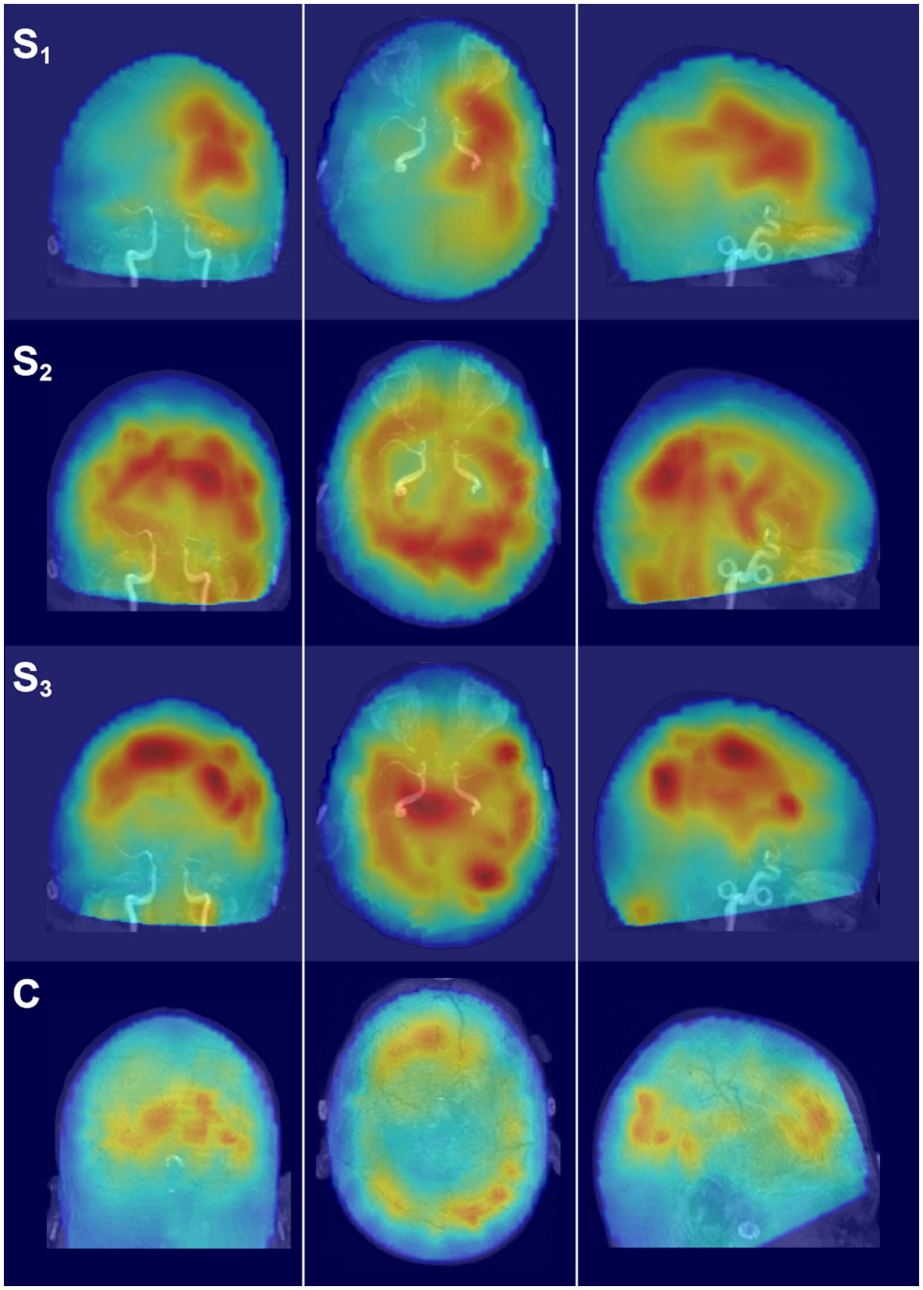
Glass brain SAM maps of activity changes. Each row depicts the coronal, axial, and parasagittal views of the SAM SPM plotted on the glass brain for the representative frequency band of alpha. Each row corresponds to a different scan time point for the patient (S_1_–S_3_) or for the representative control participant (C). Glass brain maps aid in the visualization of the distribution of activity throughout the brain volume. Maps are in radiological coordinates.

**Figure 6:**
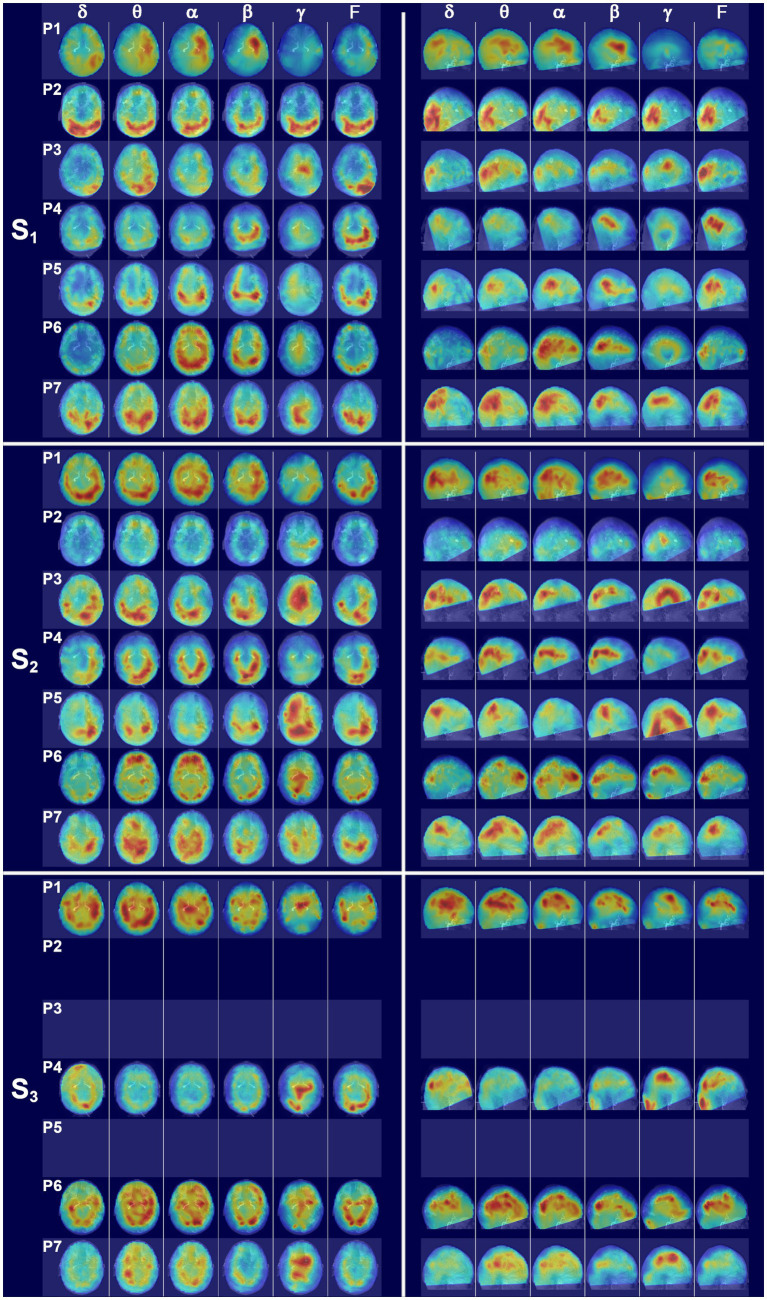
Glass brain SAM maps for all patients across scans. (S_1_) The left set of panels depicts an axial glass brain view (anterior upward) and the right panel set depicts a parasagittal glass brain view (anterior to the right) at the first scan time point for all patients across frequency bands. Coronal views were omitted for clarity. The glass brains for each patient are presented in rows and numbered according to patient, with P1 representing patient 1, etc., and the columns indicating frequency bands. (S_2_) Glass brain maps for all patients for the second scan time point. (S_3_) Glass brain maps for all patients for the third scan time point. Patients 2, 3, and 5 were unable to receive a third scan.

Dorsal brain surface MEG maps are presented in Figure 3 for scans 1, 2, and 3. Gray regions on the map indicate areas of minimal activity for a particular bandwidth. As the patient recovered, activity increased in the right hemisphere across all bandwidths, which was reflected in a reduction in asymmetry relative to scan 1 (Supplementary Figure 2). Gamma activity was prominent at scan 3 but was diminished or absent for scans 2 and 1, respectively. Maps for a single, representative control participant are plotted on the same color scale at the bottom of the panel to facilitate comparison. Patient 1 exhibited greater hemispheric asymmetry (in general left greater than right) at all time points relative to the control participants, although for some ROI pairs the activity in the right hemisphere far exceeded the left (Supplementary Figure 2). The mesial and lateral brain surfaces of the left and right hemispheres are presented in Figure 4 for the patient and for the control participant. At scan 3, the patient exhibited greater activity in the left temporal lobe in comparison to the damaged right temporal lobe for all frequencies above delta. Example alpha-band glass brain maps for patient 1 and for the control participant are presented in Figure 5 and depict coronal, dorsal, and parasagittal views. Figure 6 depicts the dorsal and parasagittal glass brain views for each patient (P1–7) according to frequency band and scan time point to facilitate comparisons across the group. The glass brain maps provide an estimate of the volumetric distribution of brain activity at each time point and further highlight the dearth of sources throughout the brain volume at scan 1 for the patient, the asymmetry of the patient’s source maps for scans 1 and 2, the increase in gamma across scan time points, and in combination with the other plots reveal that SAM power is elevated across bandwidths at scan 3 for the patient relative to the control participant.

### 3.2. Patient 2

Patient 2 was a 53 y.o., right-handed male with PMH of substance abuse who presented as an unrestrained driver in an MVC. He was found on scene with agonal breathing and was intubated and transferred to our facility where a head CT demonstrated multiple areas of IPH in the right and left supratentorial regions, including multiple foci in the splenium and other parts of the corpus callosum, midbrain, and bilateral subcortical regions, bilateral SDH, small volume intraventricular hemorrhage (IVH), SAH, evidence of DAI, facial (left mandibular and nasal) fractures, multiple closed rib fractures, and a closed fracture of the spinous processes of the thoracic vertebrae.

This patient received his first MEG scan 8 days after injury. At scan 1 (Figure 6, S_1_), patient 2 (P2) exhibited a large amount of power at all bandwidths, but power predominantly localized to the bilateral ventral posterior brain regions, with the left hemisphere having greater power than the right (Supplementary Figure 3). In contrast, dorsal parietal and frontal regions exhibited minimal power across bandwidths. Gamma power was also globally suppressed across brain structures, apart from bilateral occipital cortex, which had excessive power across all bandwidths.

Dorsal and parasagittal glass brain maps for scan 2 for all seven patients are presented in Figure 6, S_2_. At scan 2 (eyes closed), 52 days after injury, power was greatly reduced across all bands throughout the whole brain volume for patient 2, with a continued dearth of power in dorsal parietal and dorsal frontal regions, and a continued asymmetry with the left hemisphere having greater power than the right (Supplementary Figure 3). A modest increase in midline gamma was evident at the second time point in comparison to the first. This patient did not receive a third MEG scan.

### 3.3. Patient 3

Patient 3 was a 56 y.o., right-handed male with PMH of Lyme’s disease who presented after sustaining injuries from a ~25-foot fall onto concrete. He was intubated in the field after he was found comatose with an obvious skull deformity. His injuries included a depressed skull fracture, large right sided SDH with significant midline shift and subfalcine herniation, multiple rib, cervical and thoracic vertebral fractures, sternal fracture, anterior mediastinal hematoma, and mild dissection of the right internal carotid artery. The patient underwent urgent decompression with hemicraniectomy and evacuation of SDH.

Patient 3 received his first MEG scan 14 days after injury. His MEG scan was notable for hemispheric asymmetry (Supplementary Figure 3) with excessive delta, theta, and full bandwidth power in the left posterior cortical regions (Figure 6, S_1_) and, in particular, the left occipital and temporal cortices. Right hemispheric activity was reduced. Outside of the posterior ROIs, power was greatly reduced across dorsal and frontal regions, and midline gamma activity was weakly present. At the second scan 7 weeks later (eyes closed), source power across bandwidths was still generally distributed posteriorly for patient 3 but activity in the right posterior regions had increased (Figure 6, S_2_) relative to the first scan, although the left hemisphere still had greater activity, particularly in the delta band (Supplementary Figure 3). Gamma power had also greatly increased in dorsal midline regions relative to the first scan. Due to a cervical collar, this patient was not inserted fully into the helmet and thus had incomplete MEG coverage of ventral frontal and temporal structures. This patient did not receive a third MEG scan.

### 3.4. Patient 4

Patient 4 was a 54 y.o., right-handed male with no PMH who presented after a tree limb fell on him. He was found comatose by EMS and was intubated in the field. His injuries included traumatic SAH, fractures of the multiple levels of the cervical, thoracic, and lumbar spine, left ribs, pelvis, left tibia and fibula, and right posterior malleolus. An MRI revealed diffusion restriction in bilateral frontal and right parietal white matter areas, right forceps and cingulate, and evidence of shear injury and DAI.

Patient 4 received his first MEG scan 9 days after injury, which was notable for activity centered in the ventral and posterior cortical regions across the frequency bands, with greater left hemispheric activity than right (Figure 6, S_1_; Supplementary Figure 3). In the remaining superior and anterior cortical regions, activity was diffusely decreased across bandwidths. Gamma was globally reduced. Owing to a cervical collar, the patient was not fully inserted into the helmet and the left inferior occipital cortex and parts of the crus were not covered.

At the second scan 6 weeks later (eyes open), patient 4 had a marked increase in brain activity relative to the first scan timepoint, but the activity was still largely restricted to ventral and posterior brain regions across bandwidths, with activity greater in the left hemisphere (Figure 6, S_2_; Supplementary Figure 3). Due to the C-collar, coverage was incomplete for bilateral ventral frontal cortex and part of the anterior temporal lobes. Gamma activity remained minimal for scan 2.

This patient received a third MEG scan, with whole head coverage, 9 months after injury (Figure 6, S_3_). Glass brain maps reveal a trend toward bilateral symmetry across hemispheres with some right sided ROIs now having greater power than the left (Supplementary Figure 3). There was also a decrease in power across delta through beta relative to the second scan and a marked increase in dorsal gamma as compared to scans 1 and 2. While the power was not particularly strong, frontal and dorsal sources were present at scan 3.

### 3.5. Patient 5

Patient 5 was a 71 y.o., right-handed female with PMH of seizures, hypertension, hyperlipidemia, esophageal reflux, peptic ulcer, and elevated liver enzymes who presented to another facility after being struck by a motor vehicle as a pedestrian, falling and hitting her head. On EMS arrival the patient was reported to have been “awake and alert” but was later intubated due to declining mental status. Her injuries included bifrontal cerebral contusions, foci of IPH, SAH, predominantly left SDH, left temporal bone fracture, and multiple sinus thrombosis. The patient underwent a left decompressive hemicraniectomy with evacuations of the supratentorial SDH and frontal IPH after early, substantial worsening of her intracranial bleeds.

Glass brain MEG maps at scan 1, 3 days after injury (Figure 6, S_1_), revealed a posterior and dorsolateral concentration of activity that was roughly symmetric across hemispheres (Supplementary Figure 3). Right ventral frontal activity was present, particularly at alpha and beta, but there was a dearth of power in the left frontolateral regions consistent with the site of injury. Gamma power was minimal at the first scan, with a greater relative distribution of power in the right dorsal regions in comparison to the left.

The second MEG scan (eyes open) 12 weeks after injury was marked by increases in activity across brain regions relative to scan 1 (Figure 6, S_2_). Gamma was dramatically increased throughout the right hemisphere and posteriorly in the left. The left frontolateral regions remained consistently depressed across all frequency bands at scan 2. Owing to a hard-cervical collar, coverage was incomplete for the bases of the temporal lobes and cerebellum. This patient did not receive a third MEG scan.

### 3.6. Patient 6

Patient 6 was a 26 y.o., right-handed male with PMH of asthma who presented after sustaining injuries in an MVC. He was intubated at the scene. His injuries included left SDH with midline shift and vertebral artery dissection.

Patient 6 received his first MEG scan 3 days after injury, and the glass brain SAM maps (Figure 6, S_1_) were notable for approximate hemispheric symmetry (Supplementary Figure 3) and a posterior predominance of biomagnetic activity. Bilateral ventral frontal sources were present in the theta through beta bands. Modest gamma was visible in the dorsal midline regions. At scan 2, 3 weeks after injury and with eyes open, the glass brain maps were notable for a shift in theta and alpha power to frontal ROIs and away from the posterior brain regions in comparison to scan 1, as well as increases in dorsal and midline gamma (Figure 6, S_2_). Seven months after injury, patient 6 underwent scan 3. In comparison to scans 1 and 2 for this patient, the glass brain maps exhibited a continued increase in power across all frequency bands and across brain regions (Figure 6, S_3_). Gamma power remained strong at scan 3.

### 3.7. Patient 7

Patient 7 was a 28 y.o., left-handed male with PMH of esophageal reflux and polysubstance abuse who presented after sustaining injuries in an MVC. He was unresponsive and was intubated on the scene. His injuries included left-sided SDH and evidence of IPH, left vertebral artery dissection, fractures of the cervical and thoracic spine, ribs, pelvis, right femur and ankle, and splenic and liver lacerations.

Patient 7 received his first MEG scan 7 days after injury, and the glass brain maps (Figure 6, S_1_) revealed a posterior activation pattern with power stronger in the left than right hemisphere for all frequency bands except gamma. Gamma was concentrated posteriorly and dorsolaterally, with the right hemisphere stronger than the left (Supplementary Figure 3). At scan 2 (eyes open), 4 weeks after injury, the glass brain maps were notable for increased dorsal activity relative to scan 1 (Figure 6, S_2_). Gamma power was diffusely localized across dorsal structures. Nine months after injury, patient 7 underwent scan 3 (Figure 6, S_3_). The glass brain maps were showed a decrease in hyperactivity in posterior brain regions relative to scan 2, suggesting a normalization in brain function. Gamma was increased in dorsal and midline structures relative to scan 2.

### 3.8. Group results

**Figure 7:**
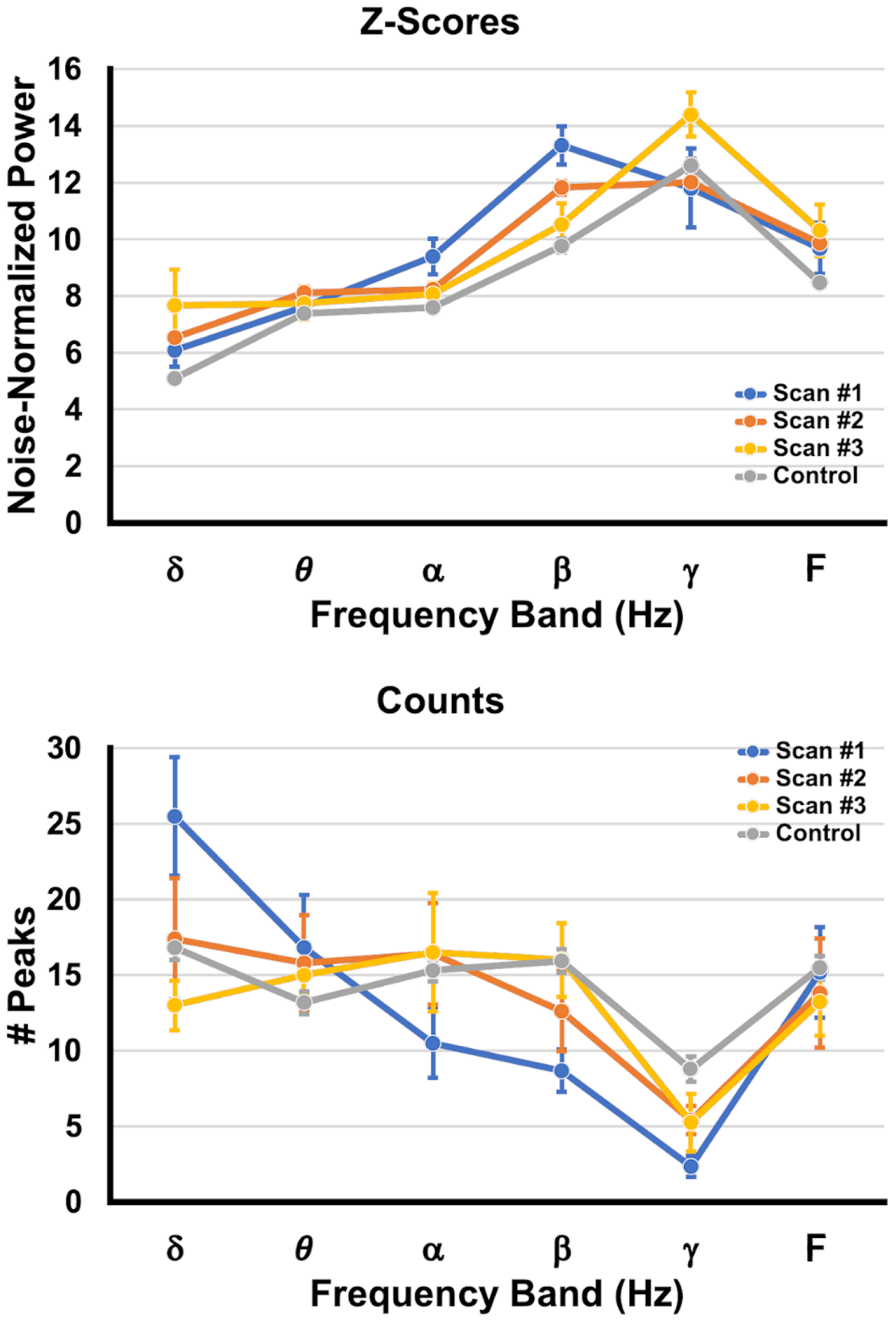
Changes in peak power and peak counts across time. The top graph (Ƶ-scores) depicts the average SAM peak Ƶ-score ± SEM (shown on the ordinate) per frequency band (abscissa), collapsed across brain regions and pooled across patients. The bottom graph (Counts) depicts the average number ± SEM of SAM peaks (generators) per frequency band, collapsed across brain regions and pooled across patients. The values for scan 1 are presented in blue, those for scan 2 are orange, those for scan 3 are yellow, and values obtained from the control group are depicted in gray.

The top panel in Figure 7 depicts the average noise-normalized power (as given by the Ƶ-score) ± SEM at each frequency band for scans 1, 2, and 3, as well as the corresponding values for the control participants. The bottom panel in Figure 7 depicts the average number of neural generators ± SEM at each frequency band for each scan as well as values for the control participants. The multiple linear regression model for the Ƶ-score values indicated significant omnibus effects for scan number, frequency band, and the interaction between scan number and frequency band, with all p’s < 0.000055. Given the significant interaction term, simple effects contrasts were performed with a Holm correction for the FWER such that the Ƶ-score power for the controls was compared to the values for each of scans 1–3 for the patients with sTBI, and within each bandwidth. The simple effects contrast revealed that there was significantly more power in the alpha and beta bandwidths for scan 1 vs. the control participants, significantly more beta power for scan 2 vs. controls, and more delta power at scan 3 vs. controls (all corrected *p*’s < 0.05).

The multiple linear regression model for the number of peaks (neural generators) indicated significant omnibus effects for frequency band and the interaction between scan number and frequency band, with all *p*’s < 0.00002. Given the significant interaction term, simple effects contrasts were performed with a Holm correction such that the number of peaks for the controls was compared to the numbers of peaks for each of scans 1–3 for the patients with sTBI, and within each bandwidth. The simple effects contrast revealed that there were significantly more peaks in the delta bandwidths for scan 1 vs. controls, and significantly fewer peaks in the beta and gamma bandwidths for scan 1 vs. the controls (all corrected *p*’s < 0.05). Scans 2 and 3 did not differ from controls in the number of peaks for each bandwidth.

## 4. Discussion

This is the first study to use MEG as a diagnostic modality in the acute, sTBI population and shows the safety and feasibility of scanning patients on life support. This study demonstrates that MEG yields unique, non-redundant data, which in combination with clinical, imaging, EEG, and neuropsychological testing can provide a more complete picture of the injury-recovery continuum in these patients. Specifically, we used MEG to longitudinally track the evolution of brain activity in seven patients with sTBI, starting at the acute time point and with follow-up scans at ~1.5 and 8 months post injury, when available, in comparison to 30 control participants obtained from a previous study (56, 63–65).

We found that while delta power was not significantly different at scan 1 relative to controls, there was a large excess of neural generators operating within the delta band at this acute time point. The elevated alpha and beta power sources were primarily distributed posteriorly and ventrolaterally, while the number of neural generators for beta and gamma were significantly reduced. In comatose patients and those with DoC, the presence of delta activity may indicate cortical lesions as well as the presence of infarcts and/or hemorrhages, while higher frequencies are attenuated in these patients (33, 34). In the chronic phase, it has been found that delta (32, 42) and theta power are increased in VS/UWS patients in comparison to MCS patients and/or healthy controls, while both groups have decreased alpha relative to controls (39). DoC patients with a predominance of delta and slow theta are less likely to survive at 6 months whereas patients with more fast theta and alpha activity have better outcomes (38). The increased alpha and beta power may represent a compensation for the reduced number of neural generators for these bands, or it may also reflect a release from inhibition from the contralateral, damaged regions (90, 91). Gamma power is less reported in EEG studies, likely owing to the attenuation of these frequencies by the tissues of the head and the potential contamination of muscle artifact (38), whereas MEG can detect neural oscillations ≥ 1 kHz (92). The shift in brain dynamics to the delta band and the reduction in the number of gamma generators (93) is consistent with the comatose state, and is likely a result of acute thalamocortical dysfunction (36, 94–98).

Scan 2 captures a transitory phase in which the patients exhibited better GCS scores and an improved clinical neurological state. Overall, brain activity normalized, with only increased beta power for scan 2 relative to controls. At scan 3 (~7.8 months post injury), all patients who returned were ambulatory and independent, and group-wise delta power had increased relative to that for controls, which may reflect ongoing secondary neurodegeneration subsequent to initial DAI (99), as focal cortical disconnection is manifested by an increase in low frequency oscillations (100). Likewise, MEG findings in patients with mTBI also reveal increases in delta power (68, 101, 102) that colocalize to white matter lesions evident on DTI (66). Finally, for both scans 2 and 3, the number of neural generators did not differ at any bandwidth in comparison to controls, suggesting a broad reactivation of neural networks needed to maintain consciousness (15, 97, 98).

At the single patient level, SAM resting state maps revealed zones of hypofunction and islands of preserved activity, while also enabling the visualization of the return in brain function over time. At scan 1, patients 1 and 3 exhibited hemispheric asymmetry across all bandwidths, with markedly reduced power on the side of injury and islands of focal contralateral power. Similarly, in patient 5, power in the damaged left lateral frontal regions was greatly reduced across all bandwidths in comparison to the contralateral side. Across the patients, spectral power was distributed more posteriorly and ventrally, with less power in the frontal and parietal regions, which would be consistent with the role of the latter regions in maintaining consciousness (29, 97, 98).

At scan 2, increased source power across bandwidths was evident for most patients, along with an increase in GCS scores. Patients 1 and 3 exhibited increased bilateral activation relative to scan 1, although the hemisphere contralateral to the main site of injury continued to have more power across bandwidths. At scan 2, patients 2 and 4 largely lacked power in dorsal frontal and parietal regions, while patient 6 exhibited increased theta and alpha power in ventral frontal regions in comparison to scan 1 but a continued reduction in dorsal power. Similarly, patient 7 exhibited a broader distribution of activity but a continued diminishment of anterior prefrontal power for scan 2 vs. 1.

Only four patients returned for a third MEG scan. All four were ambulatory and had an increase in gamma power in comparison to their prior scans. Neural activity was distributed more broadly and evenly across the hemispheres, although asymmetries were still evident in each of the patients. Notably, patients 1, 4, and 6 showed increases in delta power across scans 1–3, suggesting possible ongoing neurodegeneration subsequent to DAI. Patients 4, 6, and 7 had increased prefrontal activation at scan 3, whereas patient 1 did not. The relative hemispheric asymmetries, power imbalances within and across bands, and focal alterations, such as diminished frontal power, may correlate with each patient’s particular neuropathology and potential functional deficits. Interestingly, neuropsychological testing revealed persistent dysfunction in mood and response control domains that was not captured by either the GCS, bedside neurological assessments, or routine outcome measures like the GOSE.

The use of MEG to measure brain activity in this context offers several advantages. MEG confers the ability to perform source localization, which establishes which brain regions are active and at what frequencies, and additionally whether particular regions are hyper- or hypoactive within a given frequency band in comparison to controls. A second advantage is that MEG is unaffected by the skull defects that would cause EEG breach effects and that would compromise EEG spectral power estimates, while MEG simultaneously provides a reliable estimate of residual brain function from lesional or perilesional tissue (59). One potential concern with SAM is that beamformers cannot recover completely synchronized signals. However, this concern is physiologically implausible because it would require the perfect and unrealistic zero-lag synchronization of brain rhythms for the entire recording duration, whereas very highly correlated signals can still be recovered by SAM (78, 81), as in the case of patients with epilepsy (72, 73).

This is a pilot study that is limited by a small sample size, precluding a direct comparison between neuropsychological outcomes and MEG functional maps. In the future, we will correlate neuropsychological measures like attention, memory, and mood with individual MEG findings in a larger study. Imaging modalities like CT and MRI when repeated 6–12 months after sTBI frequently do not reveal persistent structural abnormalities. This, coupled with grossly normal assessments are a source of dissatisfaction and frustration for patients and families who are noticing ongoing concerns. MEG (with MSI and network analyses) may finally reveal the functional correlates of sustained behavioral challenges that sTBI patients face. Further, alterations in the SAM spectral maps may suggest treatment options like deep brain stimulation, transcranial magnetic stimulation, and transcranial direct-current stimulation to normalize brain rhythms. Finally, targeted behavioral interventions can be monitored with MEG scans across time to assess physiological changes in brain areas and networks.

## Data availability statement

The datasets presented in this article are not readily available because they were obtained from patients in the course of clinical care. This is because these data were obtained from patients and sharing would violate HIPPA. Requests to access the datasets should be directed to the corresponding authors.

## Ethics statement

The studies involving humans were approved by Wake Forest University School of Medicine Institutional Review Board. The studies were conducted in accordance with the local legislation and institutional requirements. Written informed consent for participation in this study was provided by the participants’ legal guardians/next of kin. Written informed consent was obtained from the individual(s) for the publication of any potentially identifiable images or data included in this article.

## Author contributions

AS: Conceptualization, Investigation, Methodology, Project administration, Resources, Validation, Visualization, Writing – original draft, Writing – review & editing. GP: Conceptualization, Investigation, Project administration, Resources, Writing – review & editing. AA: Writing – original draft. NC: Writing – original draft. CC: Investigation, Project administration, Resources, Writing – review & editing. AN: Investigation, Project administration, Writing – original draft. JR: Formal analysis, Writing – review & editing. DG: Resources, Writing – review & editing. LF: Investigation, Project administration, Writing – review & editing. DC: Investigation, Project administration, Resources, Writing – review & editing. JS-K: Conceptualization, Formal analysis, Investigation, Methodology, Project administration, Resources, Software, Validation, Visualization, Writing – original draft, Writing – review & editing.

## Supplementary material

SUPPLEMENTARY FIGURE 1 Example of local maxima in the SAM beta map. Each row depicts coronal, sagittal, and axial views of one of the three beta band peaks that formed in the first scan for patient one. The first peak (row 1) had MNI coordinates of [–36, 12, 24], a Ƶ-score of 15.18, and was located in the left inferior frontal operculum/inferior triangle. The second peak (row 2) had MNI coordinates of [–11, –22, 44], a Ƶ-score of 9.57, and was located in the left middle cingulum. The third peak (row 3) had MNI coordinates of [–40, 16, –29], a Ƶ-score of 9.02, and was located in the left superior/middle temporal pole.

SUPPLEMENTARY FIGURE 2 Changes in hemispheric asymmetry across time for patient 1. Each column depicts the percent difference in asymmetry for pairs of ROIs for each frequency band. Column 1 corresponds to scan 1, the second column is for scan 2, and the third column depicts scan 3. The average asymmetry values for the control participants are presented in the fourth column. Each bar represents the percent difference for a particular ROI pair at a given frequency band; if the power of the left ROI was greater than the right then the difference was negative, and if the power of the right ROI was greater then the difference was positive. The AAL ROI pairs are for the following structures: Precentral, Superior Frontal, Superior Orbitofrontal, Middle Frontal, Middle Orbitofrontal, Inferior Opercular Frontal, Inferior Frontal Triangle, Inferior Orbitofrontal, Rolandic Operulum, Supplementary Motor Area, Olfactory, Superior Medial Frontal, Medial Orbitofrontal, Rectus, Insula, Anterior Cingulum, Middle Cingulum, Posterior Cingulum, Hippocampus, ParaHippocampal, Amygdala, Calcarine, Cuneus, Lingual, Superior Occipital, Middle Occipital, Inferior Occipital, Fusiform, Postcentral, Superior Parietal, Inferior Parietal, SupraMarginal, Angular, Precuneus, Paracentral lobule, Caudate, Putamen, Pallidum, Thalamus, Heschl, Superior Temporal, Superior Temporal Pole, Middle Temporal, Middle Temporal Pole, Inferior Temporal.

SUPPLEMENTARY FIGURE 3 Changes in hemispheric asymmetry across time for each patient. The percent difference in ROI asymmetry as a function of frequency bandwidth and scan number is presented for each patient. The average percent difference for control participants are plotted in the far right column to enable comparison. Conventions are the same as in Supplementary Figure 2.

## Glossary

sTBI: Severe traumatic brain injury
DoC: Disorders of consciousness
VS/UWS: Vegetative state/unresponsive wakefulness state
MCS: Minimally conscious state
IMPACT: International Mission for Prognosis and Analysis of Clinical Trials in TBI
CRASH: Corticosteroid Randomization After Significant Head Injury
DAI: Diffuse axonal injury
MEG: Magnetoencephalography
MSI: Magnetic source imaging
SAM: Synthetic aperture magnetometry
MVC: Motor vehicle collision
GCS: Glasgow Coma Scale
GOSE: Glasgow Outcome Score-Extended
SPM: Statistical parametric map
ROI: Region of interest
FWER: Family-wise error rate
PMH: Past medical history
IPH: Intraparenchymal hemorrhage
EDH: Epidural hematoma
SDH: Subdural hematoma
SAH: Subarachnoid hemorrhage
IVH: Intraventricular hemorrhage
