# Supplementary figures and images for: Use of magnetic source imaging to assess recovery after severe traumatic brain injury—an MEG pilot study
Source: Front Neurol. 2023 Nov 3;14:1257886. doi: 10.3389/fneur.2023.1257886 (PMC10656620; doi:10.3389/fneur.2023.1257886)

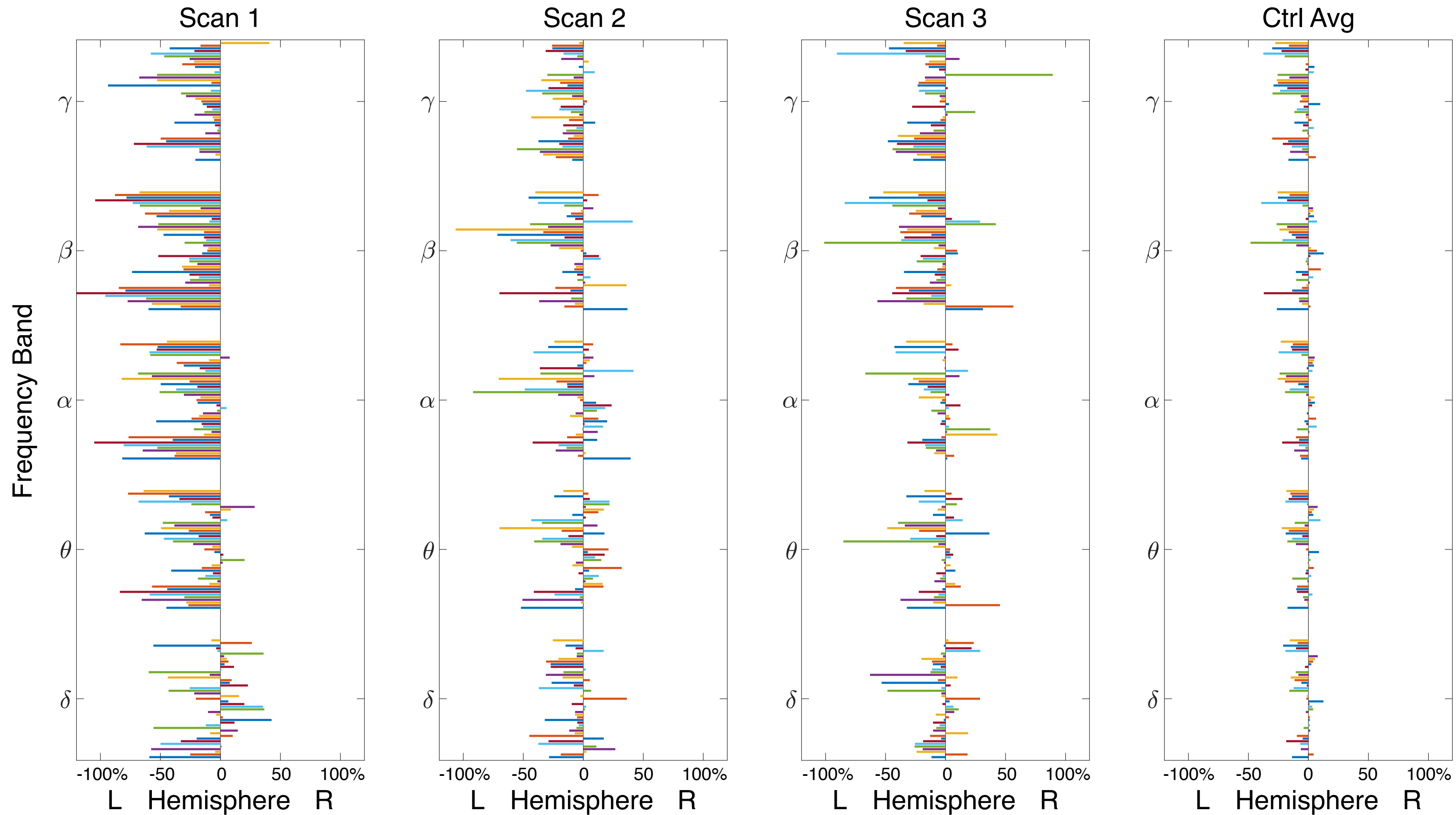

Supplement: SUPPLEMENTARY FIGURE 1 — Example of local maxima in the SAM beta map. Each row depicts coronal, sagittal, and axial views of one of the three beta band peaks that formed in the first scan for patient one. The first peak (row 1) had MNI coordinates of [–36, 12, 24], a Ƶ-score of 15.18, and was located in the left inferior frontal operculum/inferior triangle. The second peak (row 2) had MNI coordinates of [–11, –22, 44], a Ƶ-score of 9.57, and was located in the left middle cingulum. The third peak (row 3) had MNI coordinates of [–40, 16, –29], a Ƶ-score of 9.02, and was located in the left superior/middle temporal pole. [file Data_Sheet_1.PDF]

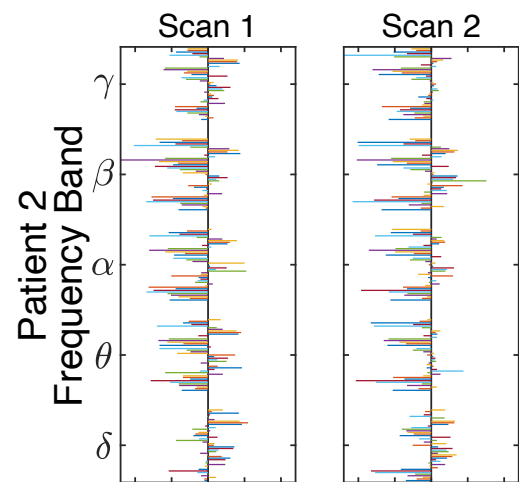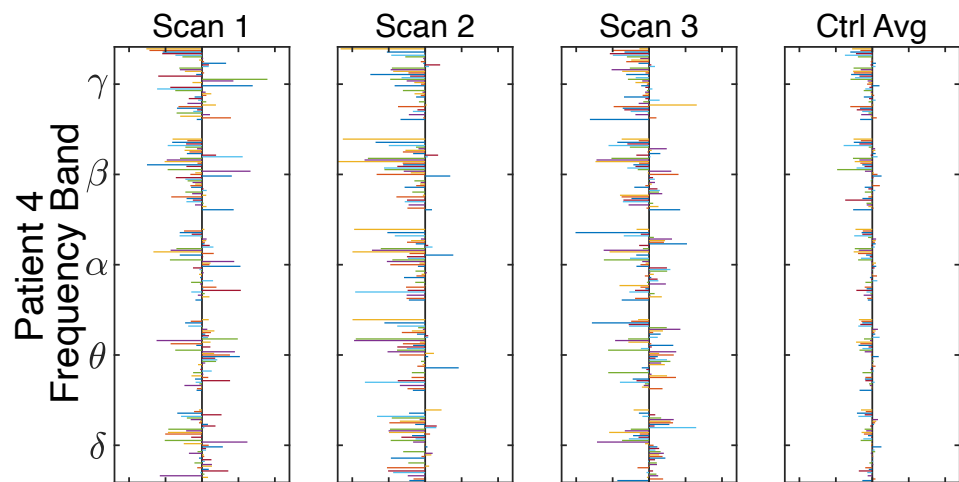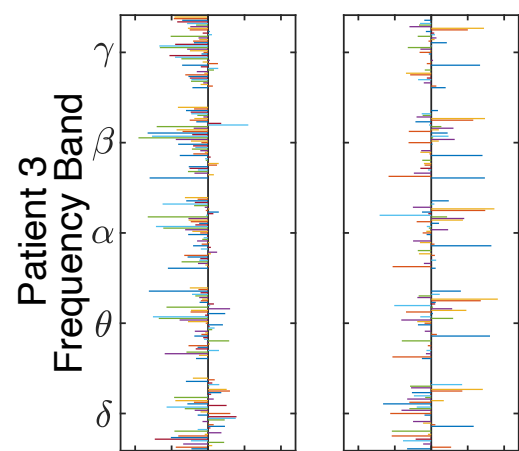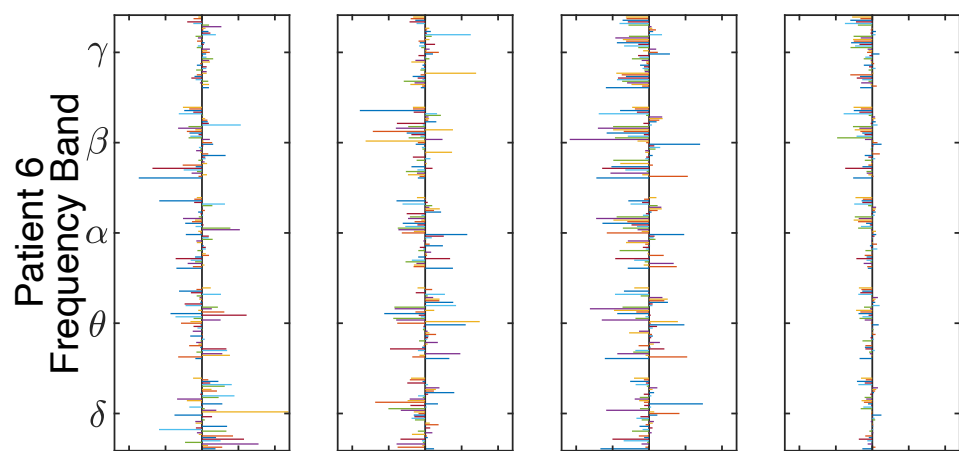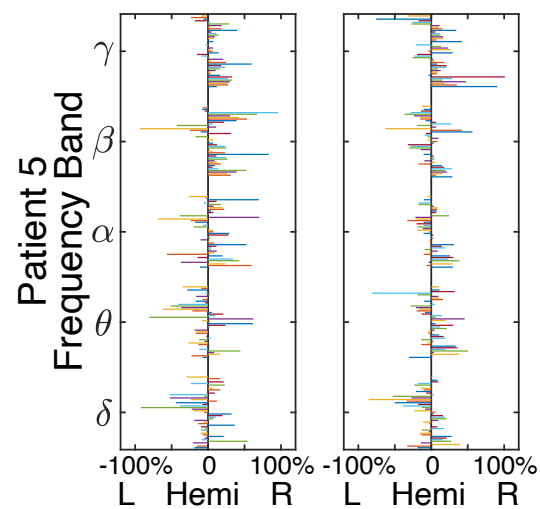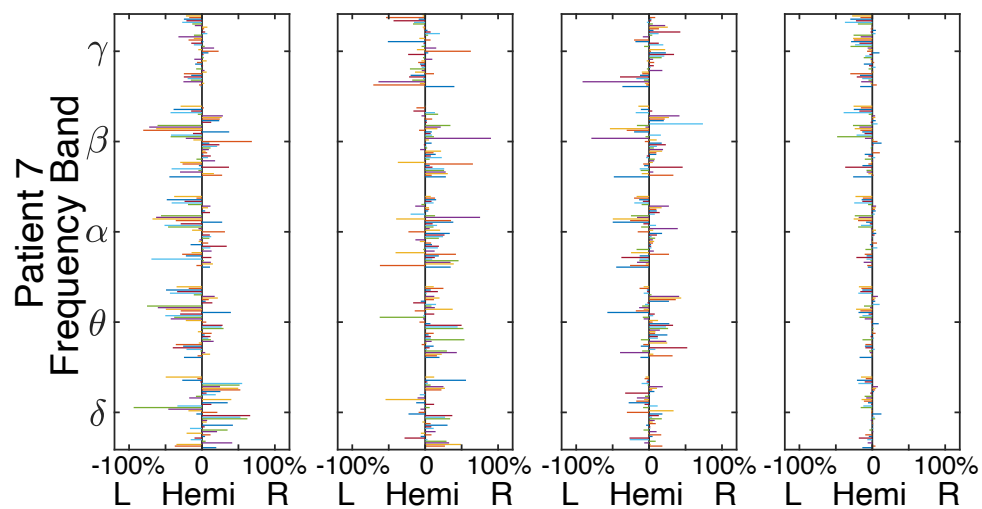

Supplement: SUPPLEMENTARY FIGURE 2 — Changes in hemispheric asymmetry across time for patient 1. Each column depicts the percent difference in asymmetry for pairs of ROIs for each frequency band. Column 1 corresponds to scan 1, the second column is for scan 2, and the third column depicts scan 3. The average asymmetry values for the control participants are presented in the fourth column. Each bar represents the percent difference for a particular ROI pair at a given frequency band; if the power of the left ROI was greater than the right then the difference was negative, and if the power of the right ROI was greater then the difference was positive. The AAL ROI pairs are for the following structures: Precentral, Superior Frontal, Superior Orbitofrontal, Middle Frontal, Middle Orbitofrontal, Inferior Opercular Frontal, Inferior Frontal Triangle, Inferior Orbitofrontal, Rolandic Operulum, Supplementary Motor Area, Olfactory, Superior Medial Frontal, Medial Orbitofrontal, Rectus, Insula, Anterior Cingulum, Middle Cingulum, Posterior Cingulum, Hippocampus, ParaHippocampal, Amygdala, Calcarine, Cuneus, Lingual, Superior Occipital, Middle Occipital, Inferior Occipital, Fusiform, Postcentral, Superior Parietal, Inferior Parietal, SupraMarginal, Angular, Precuneus, Paracentral lobule, Caudate, Putamen, Pallidum, Thalamus, Heschl, Superior Temporal, Superior Temporal Pole, Middle Temporal, Middle Temporal Pole, Inferior Temporal. [file Data_Sheet_2.PDF]

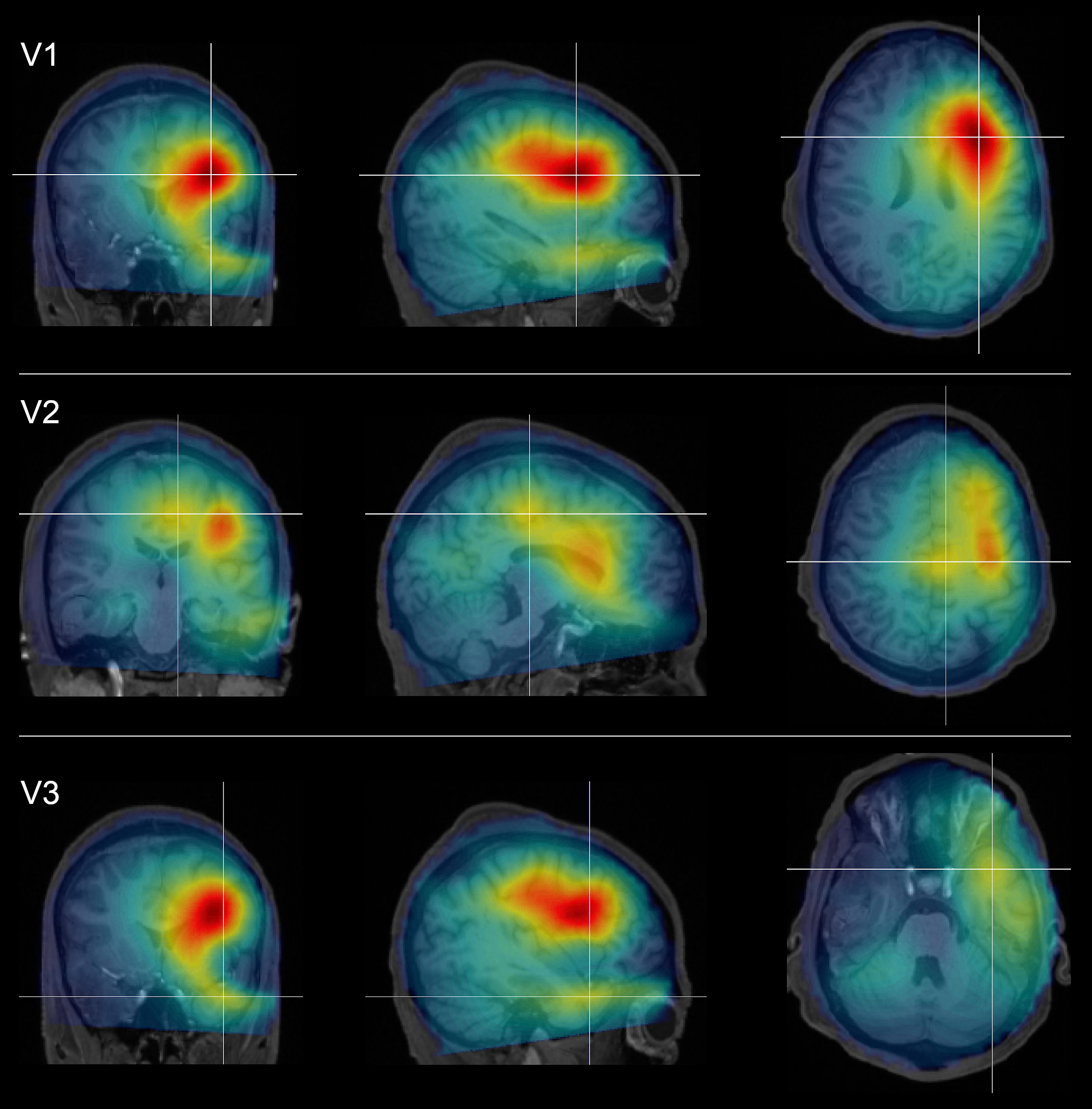

Supplement: SUPPLEMENTARY FIGURE 3 — Changes in hemispheric asymmetry across time for each patient. The percent difference in ROI asymmetry as a function of frequency bandwidth and scan number is presented for each patient. The average percent difference for control participants are plotted in the far right column to enable comparison. Conventions are the same as in Supplementary Figure 2. [file Image_1.JPEG]
